# Investigating the Inactivation Mechanism of *Bacillus subtilis* Spores by High Pressure CO_2_

**DOI:** 10.3389/fmicb.2016.01411

**Published:** 2016-09-07

**Authors:** Lei Rao, Feng Zhao, Yongtao Wang, Fang Chen, Xiaosong Hu, Xiaojun Liao

**Affiliations:** ^1^Beijing Advanced Innovation Center for Food Nutrition and Human Health, College of Food Science and Nutritional Engineering, China Agricultural UniversityBeijing, China; ^2^National Engineering Research Center for Fruit and Vegetable ProcessingBeijing, China; ^3^Key Lab of Fruit and Vegetable Processing, Ministry of AgricultureBeijing, China

**Keywords:** high pressure CO_2_, inactivation, *Bacillus subtilis* spores, mechanism, inner membrane damage

## Abstract

The objective of this study was to investigate the inactivation mechanism of *Bacillus subtilis* spores by high pressure CO_2_ (HPCD) processing. The spores of *B. subtilis* were subjected to heat at 0.1 MPa or HPCD at 6.5-20 MPa, and 64-86°C for 0-120 min. The germination, the permeability of inner membrane (IM) and cortex, the release of pyridine-2, 6-dicarboxylic acid (DPA), and changes in the morphological and internal structures of spores were investigated. The HPCD-treated spores did not lose heat resistance and their DPA release was lower than the inactivation, suggesting that spores did not germinate during HPCD. The flow cytometry analysis suggested that the permeability of the IM and cortex of HPCD-treated spores was increased. Furthermore, the DPA of the HPCD-treated spores were released in parallel with their inactivation and the fluorescence photomicrographs showed that these treated spores were stained by propidium iodide, ensuring that the permeability of IM of spores was increased by HPCD. The scanning electron microscopy photomicrographs showed that spores were crushed into debris or exhibited a hollowness on the surface, and the transmission electron microscopy photomicrographs exhibited an enlarged core, ruptured and indistinguishable IM and a loss of core materials in the HPCD-treated spores, indicating that HPCD damaged the structures of the spores. These findings suggested that HPCD inactivated *B. subtilis* spores by directly damaging the structure of the spores, rather than inducing germination of the spores.

## Introduction

Spores of a number of *Bacillus and Clostridium* species are extremely resistant to a variety of severe stresses including extreme temperatures (steam at 121°C), desiccation, chemicals and radiation because of their unique structures (Setlow, 1995, 2006). These spores are common agents of food spoilage, foodborne illnesses, and detrimental changes to the organoleptic quality of food (Brown, 2000; Logan, 2012), which make them a significant problem in the food industry. Consequently, there is much interest in methods that inactivate these spores as well as the inactivation mechanisms.

Traditionally, spores are inactivated by heat at extremely high temperature (121°C or higher) (Block, 2001). It is known that heat inactivates spores by damaging one or more proteins, most likely some enzymes involved in metabolism (Coleman et al., 2007, 2010). However, the identity of this key protein or proteins is not known. Although high temperature can effectively inactivate spores, it also can impart undesirable organoleptic changes and cause some detrimental effects to the nutritional quality of heat-sensitive food. Consequently, non-thermal technologies such as irradiation, pulsed electric fields, pulsed magnetic fields, high hydrostatic pressure (HHP), and high pressure CO_2_ (HPCD), have been proposed as food-processing methods. Among these technologies, the HHP is the most studied, and shows the potential to inactivate bacterial spores when combined with mild temperatures (Black et al., 2007a; Reineke et al., 2013b). However, the large investment cost due to the extremely high processing pressure and the non-continuous nature of the process hamper the industrial applications and commercialization of the HHP (Devlieghere et al., 2004; Estrada-Girón et al., 2005; Garcia-Gonzalez et al., 2007; Perrut, 2012).

The inactivation effect of HPCD was first shown in 1951 on *Escherichia coli* (Fraser, 1951). In recent years, HPCD treatment has been proposed as an alternative non-thermal pasteurization technique for foods because of its environmentally benign nature (CO_2_ is nontoxic), as well as the much lower pressure (generally lower than 30 MPa) compared with the high pressure (100-600 MPa) employed in the HHP processing (Garcia-Gonzalez et al., 2007). Previous studies indicate that HPCD at less than 30 MPa and at 20 to 40°C can effectively inactivate the vegetative forms of pathogenic and spoilage bacteria, yeasts, and molds, but has no effect on bacterial spores (Spilimbergo and Bertucco, 2003; Damar and Balaban, 2006; Zhang et al., 2006b; Garcia-Gonzalez et al., 2007; Perrut, 2012; Rao et al., 2015a). Several studies suggested that cycled-pressure HPCD or HPCD at temperature ≥60°C can effectively inactivate bacterial spores, and different inactivation mechanisms have been proposed (Spilimbergo et al., 2002; Spilimbergo and Bertucco, 2003; Spilimbergo et al., 2003; Bae et al., 2009).

One possible inactivation mechanism is that the spores are first activated and germinated, and then inactivated during the HPCD treatment. As reported in a previous study (Spilimbergo et al., 2002), a tyndallization effect (approximately 3.5-log reduction) is observed in *Bacillus subtilis* spores as a result of cycled-pressure (30 cycles/h, ΔP = 8 MPa) HPCD treatment at 15 MPa and 36°C for 30 min, and the inactivation mechanism is explained as follows: the initial pressure cycles induce spore activation such that germination takes place during the holding time between two different cycles. The germinated spores are inactivated during the cycles that follow (Spilimbergo et al., 2002). It is hypothesized that a combined treatment of temperature (at least 60°C) and CO_2_ induces shock in the spore structure, which leads to their activation. The spores start their germination during a long contact time of HPCD treatment. The geminated spores became more sensitive to the antimicrobial effect of CO_2_, which ultimately results in their inactivation (Spilimbergo and Bertucco, 2003; Spilimbergo et al., 2003). However, there is no data to support the claim that the spores are truly germinated during HPCD treatment in these studies. Although it has been reported that 40% of *Bacillus coagulans* spores and 70% of *Bacillus licheniformis* spores are germinated by HPCD at 35°C for 120 min (Furukawa et al., 2004), it is not clear whether the spores can be induced to germinate by HPCD at temperature ≥60°C.

Another possible inactivation mechanism is that HPCD inactivates spores by directly damaging the spore structures. A previous study used scanning electron microscopy (SEM) and energy-filtering transmission electron microscopy (EF-TEM) methods to investigate the morphological changes of *Alicyclobacillus acidoterrestris* spores treated by HPCD at 10 MPa and 70°C for 30 min (Bae et al., 2009). The SEM photomicrographs revealed that the treated spores were crushed and exhibit a high degree of hollowness on the surface. The EF-TEM photomicrographs showed an enlarged periplasmic space and a loss of cytoplasm in the treated spores. Based on these images, the authors concluded that HPCD directly affected and inactivated the *A. acidoterrestris* spores (Bae et al., 2009). However, the germination of spores was not examined in the study, and there are no data to exclude the possibility that spores may first germinate, and later be inactivated by damage to their structures. Moreover, it is also not clear how HPCD damages the spore structures, which needs further research. Recently, the flow cytometry method (FCM) has been used to assess the structural changes of high pressure treated spores stained with SYTO 16 and propidium iodide (PI) (Mathys et al., 2007; Reineke et al., 2013a). It is reported that the dormant and decoated spores are not stained by SYTO16. However, after hydrolysis of the spore cortex, the membrane-permeable SYTO 16 can permeate into the spore core and exhibit green fluorescence by binding to the nucleic acid (Black et al., 2005; Black et al., 2007b). Therefore, SYTO 16 can be designed as an indicator of the damage of the spores’ cortex. Meanwhile, PI red fluorescence nucleic acid dye is membrane impermeable, and used to identify the damage of the spores’ inner membrane (IM) (Mathys et al., 2007).

In our previous work, *B. subtilis* spores were inactivated by HPCD at temperatures higher than 82°C, and exhibited non-linear inactivation curves with a shoulder and a log-linear region (Rao et al., 2015b). The inactivation included two steps as follows: the spores first lost their resistance at the shoulder regions, and then were inactivated at the log-linear regions. The loss of resistance of the spores at the shoulder regions was explained as follows: (i) spores were induced to germinate by HPCD and lost resistance; (ii) spore structures including cortex and IM as well as some important proteins crucial to spore germination and outgrowth were damaged by HPCD. Therefore, more work is needed to determine the real reasons. In the current work, we examined the spore germination by determining the loss of heat resistance (Furukawa et al., 2004; Shah et al., 2008) and the pyridine-2, 6-dicarboxylic acid (DPA) release of HPCD-treated spores (Yi and Setlow, 2010; Setlow et al., 2016). We have also investigated the permeability of the cortex and IM of spores by the flow cytometry method (FCM) and confocal laser scanning microscopy (CLSM), as well as the release of the core material of DPA. Moreover, we have visually demonstrated changes to the surface and internal structure of spores by SEM and TEM.

## Materials and Methods

### Strain and Spore Preparation

*Bacillus subtilis* 168 was obtained from the China General Microbiological Culture Collection Center (Beijing, China), and the sporulation was carried out as previously described (Rao et al., 2015b). Overnight cultures of *Bacillus* strain grown in nutrient broth were transferred to sporulation agar plates, nutrient agar containing 50 μg/mL Mn^2+^. After 1 week incubation at 37°C, the spores were harvested in a sterile flask by flushing the surface of the culture with sterile distilled water and scrapping the surface with sterile glass microscope slide. The spores collected were washed three times by centrifugation at 7,000 × *g* and 4°C for 15 min, resuspended in sterile distilled water with a concentration of approximately 10^9^ CFU/ml, and stored at 4°C until they were used. All spores (>99%) used in this work were free of growing and sporulating cells, germinated spores, and cell debris, as determined with a BX45-72P15 phase contrast microscope (Olympus, Japan). The concentration of the spore suspension was adjusted to approximately 10^7^ CFU/mL before treatments.

### HPCD Treatment

The treatment conditions were shown in **Table 1**. HPCD treatment was performed with a batch HPCD system (Liao et al., 2007). For each experiment, 20 mL of the spores suspended in sterile distilled water was transferred to a 50 mL sterile glass tube and the tube was covered with a plastic film with a 0.22 μm membrane filter in the center of aeration to prevent microbial contamination. As the pressure vessel of the HPCD system reached the experimental temperature (64-66°C or 84-86°C), the sample tubes were placed in the pressure vessel. Next, the vessel was pressurized by the plunger pump to 6.5 or 20 MPa within 0.1 or 2.5 min, respectively. After holding for required treatment time, the depressurization was performed by opening the pressure relief valve at the CO_2_ outlet on the pressure vessel. The depressurization time was 0.5 min or 2.5 min for 6.5 or 20 MPa, respectively. After HPCD, the sample tubes were taken out from the vessel and analyzed immediately. The CO_2_ purity was 99.5% in all the experiment treatments.

**Table 1:** High pressure CO_2_ (HPCD) treatment conditions for different figures.

	Temperature (°C)	Pressure (MPa)	Holding time (min)	Pressurization (min)	Depressurization (min)
**Figure 1A**	64-66	6.5	0-60	0.1	0.5
**Figure 1B**	64-66	20	0-60	2.5	2.5
**Figure 1C**	84-86	6.5	0-30	0.1	0.5
**Figure 1D Figures 2–7**	84-86	20	0-30	2.5	2.5

The inactivation of the spore suspensions by heat at 86°C was carried out at 0.1 MPa without the addition of CO_2_ using a water bath. Similar to HPCD treatment, 20 mL of the spore suspension was transferred to a 50 mL sterile glass tube and immersed in a water bath equilibrated at 86°C for 0-30 min. The experiment was done in triplicate. After treatment, the sample tubes were taken out and immediately analyzed.

### Enumeration of Surviving Spores

The number of surviving spores was determined by the viable plate count method. Each sample was serially (1:10) diluted with sterile distilled water and pour-plated on nutrient agar in duplicate. The plates were incubated at 37°C for 24 h. After incubation, the colonies were counted.

### Measurement of Germination

As it was known that the spores would lose heat resistance and release almost all the DPA after germination (Setlow, 2003), the germination of spores during HPCD treatment was investigated by determining the loss of heat resistance and DPA release (Shah et al., 2008; Setlow et al., 2016). The spores treated by HPCD were subjected to wet heat at 80°C for 20 min, then diluted and pour-plated on nutrient agar. Following incubation at 37°C for 24 h, the colonies were counted. Germination was expressed as the change in colonies before and after exposure to heat.

The DPA release was measured using the fluorescence method (Hindle and Hall, 1999). The treated spores were centrifuged at 7,000 × *g* and 4°C for 10 min, and assaying DPA in the supernatant fluid was carried out by its fluorescence with Tb^3+^ in a 96-well plate. One hundred microliter of supernatant fluid were added to 100 μL 20 μmol/L terbium (III) chloride hexahydrate buffered with 1 mol/L acetic acid at pH 5.6. All the samples were analyzed with a microplate reader (Multiskan MK3, Thermo, USA). Samples were excited at 270 nm, and emission spectra were collected at 545 nm. The total amount of DPA in each individual batch was determined after autoclaving at 121°C for 20 min (Zhang et al., 2006a), which was used as a positive control while the one in untreated spores was used as a negative control. HPCD-induced DPA release was calculated by the following equation:

(1) $$\text{DPA}\%=\frac{{\text{F}}_{1}-{\text{F}}_{0}}{{\text{F}}_{2}-{\text{F}}_{0}}$$

Where F_0_, F_1_, and F_2_ were the fluorescence intensity of untreated spores, HPCD treated spores, and autoclaved spores, respectively.

### Flow Cytometry Analysis

Samples for flow cytometry were prepared with two DNA staining dyes according to a reported method (Reineke et al., 2013a). The dyes propidium iodide (PI) (Sigma–Aldrich) and SYTO 16 (Invitrogen) are both able to stain DNA. The membrane-permeable SYTO 16 acts as an indicator for cortex damage (Black et al., 2005), and the membrane-impermeable PI indicates the IM damage (Mathys et al., 2007). The treated spore suspensions were adjusted to concentrations of about 10^7^ spores/mL in sterile distilled water. The concentration of the fluorescent dyes in the spore suspensions were 15 μmol/L PI and 0.5 μmol/L SYTO 16. Afterward, the samples were stored in the dark at room temperature for 45 min (Pappas et al., 2015).

Stained samples were then analyzed with an Accuri C6 (BD Accuri Cytometer Inc., USA) flow cytometry equipped with a 488 nm, 50 mW laster. SYTO 16 fluorescence was quantified with the FL1 detector at 530 ± 15 nm. PI fluorescence was quantified with the FL2 detector at 585 ± 20 nm. The forward scatter threshold was set at 5000 to ensure that the small spores were not omitted as events. Spores were analyzed at a nominal flow rate of 14 μL/min, with a stream core diameter of 10 μm. All samples were evaluated after 30000 events had been recorded. The live gate, in which spores are all alive and cannot be stained with PI or SYTO16, was based on untreated spores as negative controls, while the dead gate, where the spores are all dead and can be stained with PI or SYTO16, was based on the spores exposed to 121°C for 20 min as positive controls.

### Fluorescence Analysis

The untreated, autoclaved (121°C, 20 min), heat treated (86°C, 20 min), and HPCD treated, spore suspensions were adjusted to about 10^7^ spores/mL in sterile distilled water and stained with 15 μmol/L PI for 45 min at room temperature. The stained spore samples were imaged with a Zeiss LSM710 confocal laser scanning microscope (Zeiss, Germany) with 100× oil lens. The fluorescent photomicrographs were acquired with the Zeiss AIM image browser software (Zeiss, Germany).

### Spore Preparation for SEM and TEM

For both SEM and TEM analysis, spore suspensions were centrifuged (10,000 × *g*) and prefixed in 2.5% glutaraldehyde (Sigma–Aldrich) overnight at room temperature, rinsed three times in 0.1 mol/L phosphate-buffered saline (PBS) for 15 min and centrifuged again (10,000 × *g*). The spore pellets were postfixed in 1% osmium tetroxide (Sigma–Aldrich) for 90 min and rinsed three times in 0.1 mol/L PBS for 15 min, and subsequently dehydrated with ethanol series (50, 70, 80, 90 and 100%). For SEM, the dehydrated spore samples were stored at -20°C for 20 min and subjected to critical point drying. The spore samples were sputter coated in about 12 nm of gold and palladium under vacuum, and subsequently analyzed by SEM (FEI Quanta 200, FEI, Czech Republic). For TEM, the dehydrated spore samples were embedded in epoxy resin and kept at 37°C overnight followed by 60°C for 24 h. The resin blocks were cut into ultrathin sections of 70 nm with an ultramicrotome (Lecia EM UC6, Leica, Germany) and stained with 3% aqueous lead citrate and 3% aqueous uranyl acetate. Finally, the spore samples were examined by TEM (H-7650B, Hitachi, Japan).

### Data Analysis

Flow cytometry data were analyzed using the FlowJo version 7.6.1 software (FlowJo). Analysis of variance (ANOVA) was carried out by using software PASW statistic 18 (SPSS, USA). ANOVA tests were carried out for statistical significance of group differences at α = 0.05 level. All experiments were carried out in triplicate.

## Results

### Germination Detection

**FIGURE 1:**
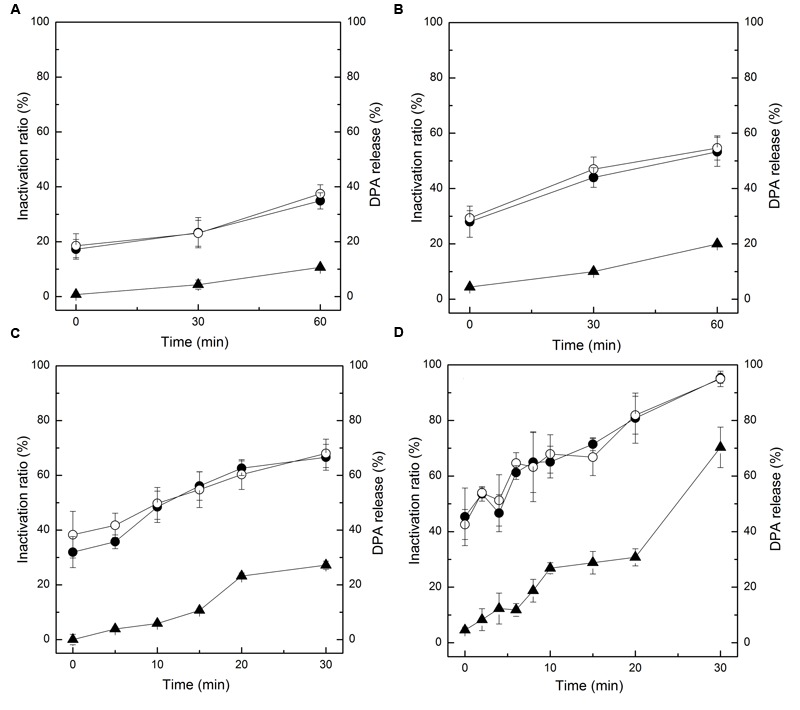
**Loss of heat resistance and pyridine-2, 6-dicarboxylic acid (DPA) release of *Bacillus subtilis* spores by high pressure CO_2_ (HPCD) at different conditions. (A)** 6.5 MPa, 64-66°C, 0-60 min; **(B)** 20 MPa, 64-66°C, 0-60 min; **(C)** 6.5 MPa, 84-86°C, 0-30 min; **(D)** 20 MPa, 84-86°C, 0-30 min. (●) spore inactivation by HPCD; (○)spore inactivation by HPCD + 80°C, 20 min; (▲) DPA release of spores treated by HPCD.

**Figures 1A–D** show the germination of the HPCD-treated spores estimated by the loss of heat resistance. The inactivation of the HPCD-treated spores increased with increasing time. After HPCD treatment at the temperature of 64-66°C for 30 min (**Figures 1A,B**), the inactivation was 23.3% at 6.5 MPa and 44.0% at 20 MPa. When the temperature was increased to 84-86°C (**Figures 1C,D**), the inactivation was increased to 66.6% at 6.5 MPa and 95.2% at 20 MPa. After all the HPCD-treated spores were subjected to heat treatment at 80°C for 20 min, there were no changes in the inactivation of the treated spores (*P* > 0.05), indicating that there were no germinated spores in the HPCD-treated spores. The DPA release of the HPCD-treated spores was also determined. Similarly, the DPA release was increased with increasing the time. After HPCD treatment at 64-66°C for 30 min (**Figures 1A,B**), the DPA release was 4.3% at 6.5 MPa and 10.0% at 20 MPa. When the temperature was increased to 84-86°C (**Figures 1C,D**), the DPA release was 27.2% at 6.5 MPa and 70.3% at 20 MPa. Obviously, the DPA release was much lower than the corresponding inactivation (*P* < 0.05), suggesting that a portion of the inactivated spores, rather than all of them, released the DPA.

### FCM Analysis

**FIGURE 2:**
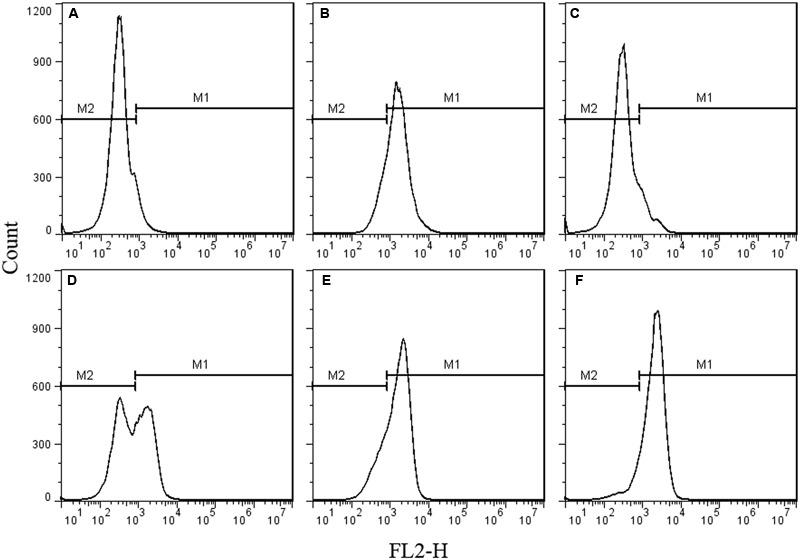
**Flow cytometry histograms of *B. subtilis spores* treated by HPCD at 20 MPa, 84-86°C for 0-20 min and stained by propidium iodide (PI). (A)** untreated; **(B)** autoclaved at 121°C for 20 min; **(C)** heat treated at 86°C for 20 min; HPCD treated at 20 MPa, 84-86°C for 0 min **(D)**, 10 min **(E)**, 20 min **(F)**.

**FIGURE 3:**
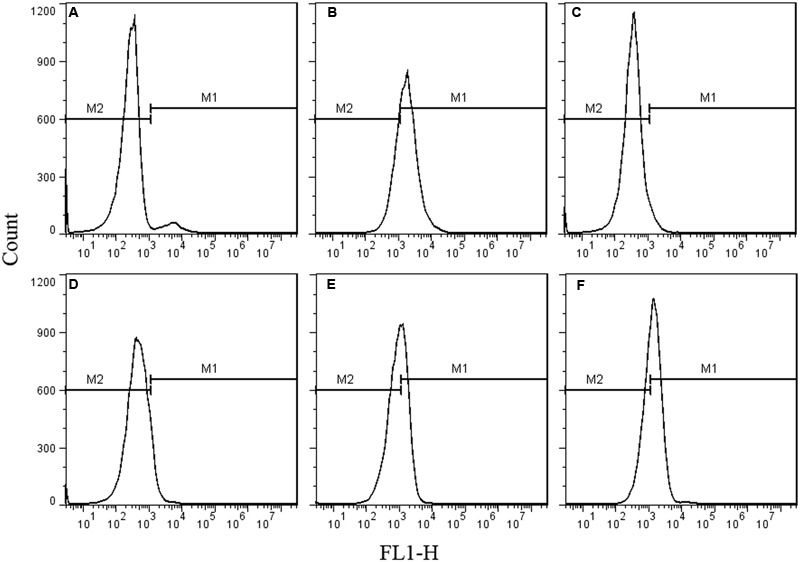
**Flow cytometry histograms of *B. subtilis spores* treated by HPCD at 20 MPa, 84-86°C for 0-20 min and stained by SYTO 16. (A)** untreated; **(B)** autoclaved at 121°C for 20 min; **(C)** heat treated at 86°C for 20 min; HPCD treated at 20 MPa, 84-86°C for 0 min **(D)**, 10 min **(E)**, 20 min **(F)**.

As the spores were not germinated during the inactivation process by HPCD, the spores were presumed to be inactivated by damage to their structures. The permeability of the IM (**Figure 2**) and cortex (**Figure 3**) of spores were tested by FCM. The untreated spores were used as negative control, and the heat-treated spores at 121°C for 20 min (>7 log-reduction) were used as positive control. Meanwhile, the heat-treated spores at 86°C for 20 min (0.067 log-reduction) were used to do the comparison analysis with the HPCD-treated spores at 20 MPa and 84-86°C for 0 min (0.15 log-reduction), 10 min (0.45 log-reduction), and 20 min (0.85 log-reduction). As shown in **Figure 2**, the FCM histograms of red fluorescence distribution of the spores stained by PI were divided into two areas, M1 and M2. According to the histogram of the untreated spores (**Figure 2A**), M2 was the negative area in which the spores had intact IM and were not stained by PI, while M1 was the positive area, indicating that the IM was damaged, and the spores were stained by PI. Compared with the untreated spores, the fluorescence distribution of the heat-treated spores at 121°C for 20 min moved increasingly towards M1 (**Figure 2B**), indicating that the IM of the spores were damaged. Meanwhile, there was only a very slight increase of the fluorescence in M1 for the heat-treated spores at 86°C for 20 min (**Figure 2C**), suggesting that these heat-treated spores have intact IM. For the HPCD-treated spores at 20 MPa and 84-86°C (**Figures 2D–F**), the fluorescence in M1 was increased, and the fluorescence distribution moved towards M1 with increased the time, indicating that the IM of spores was damaged during the HPCD treatment. Similar results are shown in **Figure 3**. According to the FCM histogram of green fluorescence distribution of the untreated spores (**Figure 3A**), M2 was the negative area in which the spores had intact cortex, and were not stained by SYTO 16, while M1 was the positive area, indicating that the cortex was damaged, and the spores were stained by SYTO 16. Compared with the untreated spores, the heat-treated spores at 121°C for 20 min showed a marked increase of the fluorescence in the M1 (**Figure 3B**), indicating damage to the cortex of spores. For the heat-treated spores at 86°C for 20 min (**Figure 3C**), there was no change of the fluorescence in M1, suggesting that heat treatment at 86°C for 20 min did not damage the cortex of spores. For the HPCD-treated spores at 20 MPa and 84-86°C (**Figures 3D–F**), the fluorescence distribution moved towards M1 with increased time, indicating that the cortex of spores was gradually damaged during HPCD treatment.

### DPA Release

**FIGURE 4:**
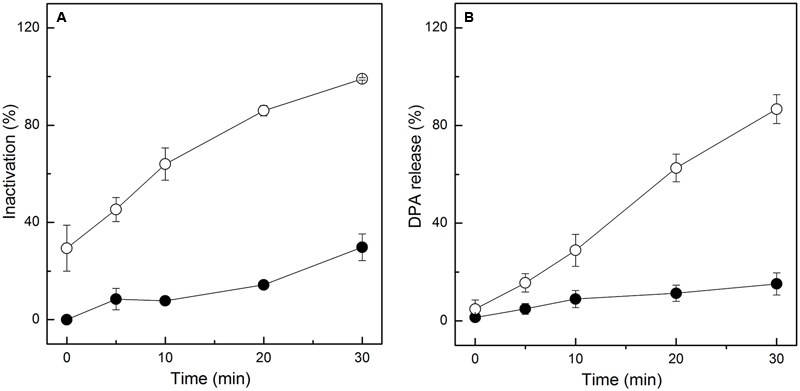
**Inactivation and DPA release of *Bacillus subtilis spores* treated by HPCD at 20 MPa, 84-86°C for 0-30 min (○) and heat at 86°C for 0-30 min (●). (A)** Inactivation; **(B)** DPA release.

Given the results of the FCM analysis, further tests were needed to ensure the damage of the IM of spores. Thus, the release of DPA, main core material in spores, was determined after HPCD treatment at 20 MPa and 84-86°C for 0-30 min. Meanwhile, the heat-treated spores treated at 86°C for 0-30 min were tested and compared with the HPCD-treated spores. With increased time, the inactivation of the HPCD-treated spores was increased, and the maximum value was 99.0% (**Figure 4A**). For the heat-treated spores, the inactivation was slowly increased with increased time, and the maximum value was 29.8% (**Figure 4A**). Similar to the results of the inactivation, the release of DPA (**Figure 4B**) for the HPCD-treated spores was also increased with increased time, and the maximum value was 86.8%, which indicated that the IM of most spores were damaged during the inactivation process by HPCD. For the heat-treated spores, the release of DPA (**Figure 4B**) was slightly increased with increased time, and the maximum value was 15.2%, suggesting that most spores maintained an intact IM.

### CLSM Photomicrographs

**FIGURE 5:**
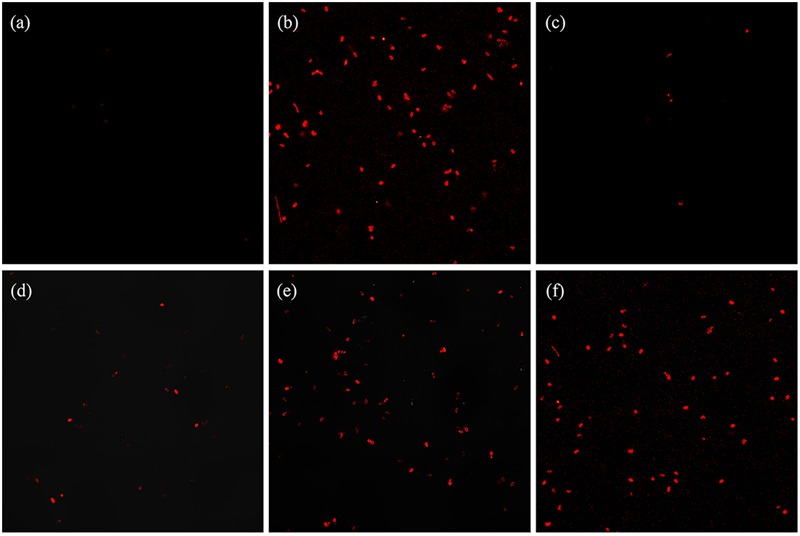
**Fluorescent photomicrographs of *B. subtilis* spores treated by HPCD at 20 MPa, 84-86°C for 0-20 min and stained by PI. (a)** untreated; **(b)** autoclaved at 121°C for 20 min; **(c)** heat treated at 86°C for 20 min; HPCD treated at 20 MPa, 84-86°C for 0 min **(d)**, 10 min **(e)**, 20 min **(f)**. The samples were imaged with 100× oil lens as detailed in the Section “Materials and Methods”.

In addition to testing the DPA release, CLSM was used to image the PI stained spores and examine the permeability of the IM of spores. The untreated spores with intact IM were not stained by PI (**Figure 5a**) while the autoclaved spores were all dead and stained red by PI (**Figure 5b**). The heat-treated (86°C, 20 min) spores (0.067 log-reduction) were not stained (**Figure 5c**), indicating these spores retained an intact IM. For the spores treated by HPCD at 20 MPa and 86°C, most of the spores were not stained or stained peripherally at 0 min (0.15 log-reduction) (**Figure 5d**) and 10 min (0.45 log-reduction) (**Figure 5e**), while completely stained at 20 min (0.85 log-reduction) (**Figure 5f**), further ensuring that the IM of the spores was gradually damaged during the HPCD treatment.

### SEM and TEM Photomicrographs

**FIGURE 6:**
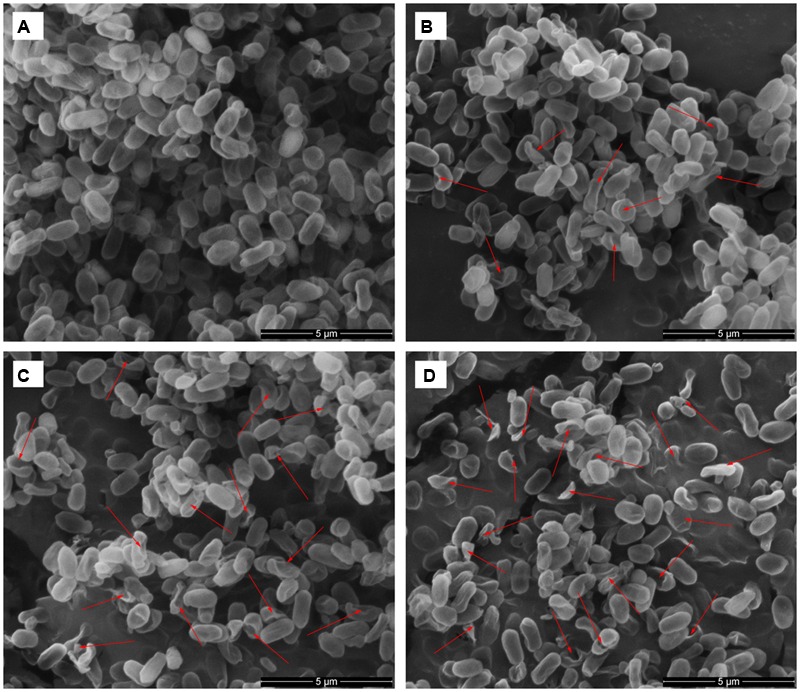
**Scanning electron microscopy photomicrographs of *B. subtilis* spores of untreated **(A)** or treated by HPCD at 20 MPa and 84-86°C for 10 min **(B)**, 20 min **(C)** and 30 min **(D)**.** Arrows indicate cell debris, and deformed or collapsed spores.

**FIGURE 7:**
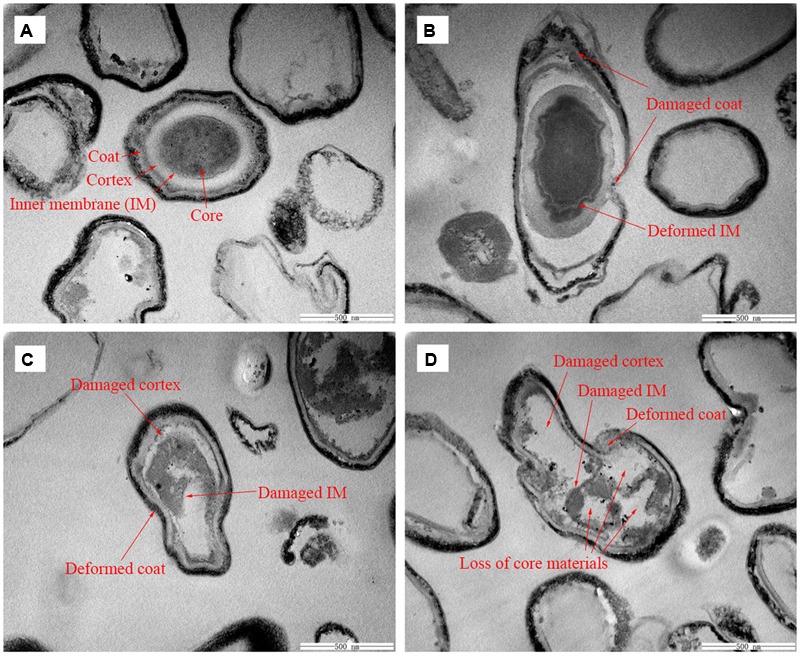
**Transmission electron microscopy photomicrographs of *B. subtilis* spores of untreated **(A)** or treated by HPCD at 20 MPa and 84-86°C for 10 min **(B)**, 20 min **(C)**, and 30 min (D)**.

To visually confirm damage to the spore structures, SEM and TEM were used to examine the morphological changes of the spores during the inactivation process by HPCD treatment at 20 MPa and 84-86°C for 10 min (**Figures 6B** and **7B**), 20 min (85.9% inactivation) (**Figures 6C** and **7C**) and 30 min (99.0% inactivation) (**Figures 6D** and **7D**). The SEM photomicrographs showed that the HPCD-treated spores were crushed into debris, or exhibited a high degree of hollowness on the surface with increased time (**Figures 6B–D**), whereas the untreated spores were intact planiform ellipsoids (**Figure 6A**). In the TEM photomicrographs, significant changes of the core area and the morphological structures of the HPCD-treated spores (**Figures 7B–D**) were observed compared with the untreated spores (**Figure 7A**). The treated spores showed an enlarged core and a loss of the core materials, and the IM of spores was ruptured (**Figures 7C,D**). Moreover, the coat of the treated spores was deformed (**Figures 7B–D**). These SEM and TEM photomicrographs further confirmed that HPCD damaged the spore structures.

## Discussion

In this study, the germination of spores was examined during the inactivation process by HPCD at different conditions (**Figures 1A–D**). As it is known that spores will lose heat resistance and release almost all the DPA after germination (Setlow, 2003; Yi and Setlow, 2010; Setlow et al., 2016), the loss of heat resistance and DPA release were used as indicators for spore germination (Shah et al., 2008; Setlow et al., 2016). Our results showed that there was no change in the population of the HPCD-treated spores after exposure to heat treatment at 80°C for 20 min (**Figures 1A–D**), suggesting that no germinated spores existed in the HPCD-treated spore population. Moreover, the DPA release was always lower than the inactivation of spores (**Figures 1A–D**), indicating that only some of the inactivated spores released DPA rather than all (Coleman et al., 2007). Thus, the inactivated spores did not undergo the germination process during the inactivation process by HPCD treatment. Indeed, there was evidence to support the premise that the spore germination may be suppressed under HPCD conditions. A previous report indicated that the germination of *Bacillus cereus, Clostridium sporogenes, and Clostridium perfringens* spores was inhibited completely by carbon dioxide at atmospheric pressure, 1.0 and 2.5 MPa, respectively (Enfors and Molin, 1978), and this inhibition of spore germination might be caused by low pH under the HPCD conditions. It was also reported that the optimum pH for pressure-induced germination at moderate pressure of 100 MPa, just like the nutrient-induced germination, was close to neutral (pH 7.0), and lowering the pH would strongly inhibit the germination (Sale et al., 1970; Bender and Marquis, 1982; Wuytack and Michiels, 2001). Another study found that reduction of pH from 7 to 5.5 completely inhibited spore germination of *Clostridium botulinum* 12885A (Blocher and Busta, 1985a). Similar results were reported for the *C. botulinum* and *B. cereus* spores at pH 4.5 (Blocher and Busta, 1985b), *B. cereus* and *B. subtilis* spores at pH 3.6 (Yi and Setlow, 2010). It was assumed that the inhibition of the spore germination in an acidic environment was induced by the inhibition of the germinant binding to the germinant receptors (GRs), which would block the commitment step of the germination (Blocher and Busta, 1985b; Yi and Setlow, 2010). This inhibition of the germinant binding was probably due to the protonation of a function group in or near the GRs (Blocher and Busta, 1985b). In our study, the HPCD treatment was conducted in a pure water-CO_2_ system, in which the pH was a strong function of pressure, but a weak function of temperature (Meyssami et al., 1992). With increased pressure, the pH decreased to approximately 3.0 at 5.5 MPa and then remained constant (Meyssami et al., 1992). Obviously, spore germination would be inhibited at such a low pH value. Given our results and those of others, it was concluded that the inactivation of spores by HPCD was not attributed to spore germination.

As the inactivation of spores by HPCD was not due to the spore germination, the question of how HPCD inactivated spores, still remains. In fact, as the CO_2_ had smaller molecular weight and higher penetrability under the HPCD state than did the spore germinants (e g., L-valine, L-alanine, D-glucose), which were able to penetrate into to the spore cells and bind to the germinant receptors (GRs) in the IM (Setlow, 2003), the CO_2_ of HPCD could theoretically penetrate into the spores cells and act on the IM. In this study, the structural changes of spores during HPCD treatment were examined. The FCM analysis indicate that the permeability of the IM of HPCD-treated spores and cortex increased (**Figures 2** and **3**), which was due to the damage to these two structures by HPCD. This damage was confirmed by the large release of the main core material of DPA (**Figure 4**) and the CLSM photomicrographs (**Figure 5**) of spores after HPCD treatment. Moreover, the SEM and TEM photomicrographs further visually confirm the damage of the morphological structures of the HPCD-treated spores (**Figures 6** and **7**). Although our results indicated that the HPCD-inactivated spores exhibited structural damage, it was necessary to determine which specific structure was damaged to the point where it caused the spore inactivation. In the FCM results for the HPCD-treated spores at 20 MPa and 84-86°C for 0 min (29.4% inactivation) (**Figures 2D** and **3D**), the red fluorescence in M1 was significantly increased (**Figure 2D**) compared with the untreated spores (**Figure 2A**), while the green fluorescence in M1 was not changed (**Figure 3D**) compared with the untreated ones (**Figure 3A**). This indicates that the damage to the spore IM occurred prior to inactivation and the damage to the spore cortex in large portion of the spores. Therefore, the damage of the IM of spores would be the reason for spore inactivation by HPCD. Similar results were reported in previous studies. It has been reported that HPCD + 0.02% H_2_O_2_ treatment at 27.5 MPa and 40°C for 240 min effectively inactivated *Bacillus atrophaeus* (Zhang et al., 2006a) and *Bacillus anthracis* (Zhang et al., 2007) spores by >6 log-reduction. The TEM photomicrographs, DPA analysis and BacLight fluorescence results in these studies indicated that HPCD damaged the IM of spores, which allowed the penetration of H_2_O_2_ into the core and subsequent oxidation of the vital structures that caused spore death (Zhang et al., 2006a, 2007). A recent study achieved more than 6 log inactivation of *B. subtilis* spores by HPCD + 0.0035-0.0055% peracetic acid (PAA) at 9.8 MPa and 35°C for 25 min, and the authors suggested that the inactivation of the spores was induced by damaging the IM of spores (Setlow et al., 2016). It is known that HPCD has the ability to extract constituents from the cells and cell membrane, modify the structure of cell membrane and damage the proteins, especially enzymes (Garcia-Gonzalez et al., 2007; Hu et al., 2010). Thus, the lethal effect of the IM damage by HPCD to the spores could be explained as follows: (i) HPCD treatment modified and increased the permeability of the IM of spores. Then, during the spore germination (if the spores are able to germinate) and outgrowth process, the germinated spores plasma membrane, which is derived from the IM of spores, became leaky and were unable to carry out proper energy metabolism (Setlow et al., 2016); (ii) the crucial proteins related to the spore germination and outgrowth in the IM, including the GRs that recognize nutrient germinants, the SpoVA proteins essential for DPA release, and the cortex-lytic enzymes that degrade cortex peptidoglycan (Setlow, 2013), were damaged by HPCD treatment, which in turn, may have induced lethal effects in the spores (Setlow et al., 2016).

Based on our results, HPCD inactivates spores by directly damaging the structures, especially the IM, rather than inducing spore germination. However, it is still not clear how the HPCD precisely acts on and damages the IM of spores. Further studies are required to examine the changes of the properties of the IM, and to identify the specific proteins whose damage induced spore inactivation by the HPCD treatment.

## Author Contributions

LR carrying out the experiments and writing the manuscript. FZ giving advice and assistance during the experiments. YW giving advice and assistance during the experiments. FC reviewing the manuscript and giving advice on the manuscript. XH reviewing the manuscript and giving advice on the manuscript. XL designing the experiments, reviewing and revising the manuscript.

## Conflict of Interest Statement

The authors declare that the research was conducted in the absence of any commercial or financial relationships that could be construed as a potential conflict of interest.
